# Differences of Variable Number Tandem Repeats in *XRCC5* Promoter Are Associated with Increased or Decreased Risk of Breast Cancer in *BRCA* Gene Mutation Carriers

**DOI:** 10.3389/fonc.2016.00092

**Published:** 2016-04-13

**Authors:** Jian Cui, Jiangtao Luo, Yeong C. Kim, Carrie Snyder, Dina Becirovic, Bradley Downs, Henry Lynch, San Ming Wang

**Affiliations:** ^1^Department of Genetics, Cell Biology and Anatomy, College of Medicine, University of Nebraska Medical Center, Omaha, NE, USA; ^2^Department of Biostatistics, College of Public Health, University of Nebraska Medical Center, Omaha, NE, USA; ^3^Department of Preventive Medicine, Hereditary Cancer Center, Creighton University, Omaha, NE, USA

**Keywords:** Ku80, *XRCC5*, promoter, VNTR, familial breast cancer, *BRCA1*, *BRCA2*, association

## Abstract

Ku80 is a subunit of the Ku heterodimer that binds to DNA double-strand break ends as part of the non-homologous end joining (NHEJ) pathway. Ku80 is also involved in homologous recombination (HR) *via* its interaction with *BRCA1*. Ku80 is encoded by the *XRCC5* gene that contains a variable number tandem repeat (VNTR) insertion in its promoter region. Different VNTR genotypes can alter *XRCC5* expression and affect Ku80 production, thereby affecting NHEJ and HR pathways. VNTR polymorphism is associated with multiple types of sporadic cancer. In this study, we investigated its potential association with familial breast cancer at the germline level. Using PCR, PAGE, Sanger sequencing, and statistical analyses, we compared VNTR genotypes in the *XRCC5* promoter between healthy individuals and three types of familial breast cancer cases: mutated *BRCA1* (*BRCA1*^+^), mutated *BRCA2* (*BRCA2*^+^), and wild-type *BRCA1*/*BRCA2* (*BRCAx*). We observed significant differences of VNTR genotypes between control and *BRCA1*^+^ group (*P* < 0.0001) and *BRCA2*^+^ group (*P* = 0.0042) but not *BRCAx* group (*P* = 0.2185), and the differences were significant between control and cancer-affected *BRCA1*^+^ cases (*P* < 0.0001) and *BRCA2*^+^ cases (*P* = 0.0092) but not cancer-affected *BRCAx* cases (*P* = 0.4251). Further analysis indicated that 2R/2R (OR = 1.94, 95%CI = 1.26–2.95, *P* = 0.0096) and 2R/1R (OR = 1.58, 95%CI = 1.11–2.26, *P* = 0.0388) were associated with increased risk but 1R/1R (OR = 0.55, 95%CI = 0.35–0.84, *P* = 0.0196) and 1R/0R (OR = 0, 95%CI = 0–0.29, *P* = 0.0012) were associated with decreased risk in cancer-affected *BRCA1*^+^ group; 2R/1R (OR = 1.94, 95%CI = 1.14–3.32, *P* = 0.0242) was associated with increased risk in cancer-affected *BRCA2*^+^ group. No correlation was observed for the altered risk between cancer-affected or -unaffected carriers and between different age of cancer diagnosis in cancer-affected carriers. The frequently observed VNTR association with in *BRCA1*^+^ and *BRCA2*^+^ breast cancer group indicates that VNTR polymorphism in the *XRCC5* promoter is associated with altered risk of breast cancer in *BRCA1*^+^ and *BRCA2*^+^ carriers.

## Introduction

Breast cancer is the major cancer type in women. Up to 20% of breast cancer cases have familial genetic background, with multiple family members across generations affected by the disease (1). The discovery of the germline mutations in *BRCA1 and BRCA2* confirmed the presence of genetic predisposition for familial breast cancer (2–4). These genes maintain genome stability in normal cells by repairing double-strand breaks mainly through homologous recombination (HR) pathway; their mutated forms lead to genome instability and increased risk for breast cancer development (5). There are two types of DNA double-strand break repair mechanisms: non-homologous end joining (NHEJ) and HR (6). Deficiency in the HR pathway, mainly caused by *BRCA* germline mutations, is well known to increase the risk of breast cancer (7); however, it is not equally clear whether deficiency in NHEJ pathway can also increase breast cancer risk (8).

Ku is a heterodimer consisting of Ku80 encoded by *XRCC5* and Ku70 encoded by *XRCC6*. Ku recognizes DNA double-strand break ends to initiate the NHEJ pathway, and Ku can also affect the function of the HR pathway by interacting with *BRCA1* (9–13). Deletion of *XRCC5* in mice leads to increased chromosomal instability, immune deficiency, growth retardation, and cancer (14, 15). Altered expression of *XRCC5* promotes oncogenic phenotypes, including hyper proliferation and resistance to apoptosis, genomic instability, and tumorigenesis (16), and has been observed in various types of sporadic cancer, including bladder, breast, colorectal, skin, esophageal, gastric, head, and neck cancer (17–22).

**Figure 1:**
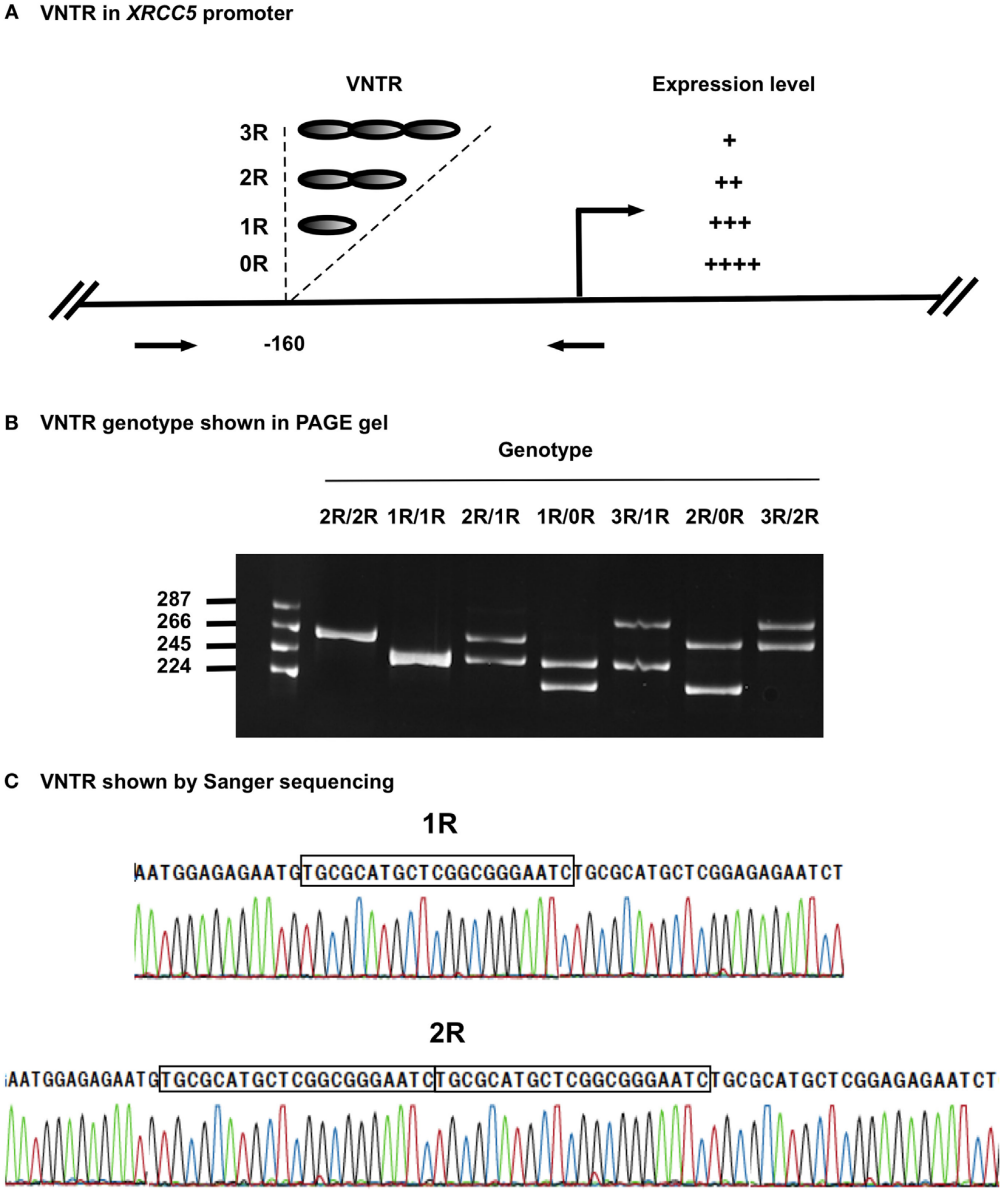
**VNTR in *XRCC5* promoter**. **(A)** VNTR types and position in the promoter of *XRCC5*. The VNTR is located at −160 bp, with 3R, 2R, 1R, and 0R alleles. Arrows refer to PCR primers used to amplify the VNTR region for genotyping. It also shows higher copies of VNTR lead to lower *XRCC5* expression (21–23). **(B)** Size distribution of different VNTR genotypes. PCR products of different genotypes were separated on an 8% PAGE gel. 2R/2R and 1R/1R had single band, other were heterozygotes with two bands, of which 2R/1R, 1R/0R, and 3R/2R had 21-base differences, and 3R/1R and 2R/0R had 42-base differences; **(C)** Sanger sequencing validation of 1R/1R and 2R/2R genotypes. It shows the 21-base unit (TGCGCATGCTCGGCGGGAATC) in 1R, and 42-base unit in 2R. 3R/3R DNA was not available for sequencing due to its rarity in human population.

Variable number tandem repeats (VNTRs) are tandem repeat DNA sequences often located in gene regulatory regions that can influence gene expression (23–25). VNTRs follow a Mendelian pattern of inheritance. The *XRCC5* promoter contains a VNTR at −160 bp, with a 21-bp repetitive unit (TGCGCATGCTCGGCGGGAATC) hosting a putative Sp1-binding site (26). Studies in Chinese and Iranian populations have demonstrated the presence of VNTR alleles ranging from 0 to 3 21-bp tandem repeats (0R, 1R, 2R, and 3R), with individual genotypes of 0R/0R, 1R/0R, 1R/1R, 2R/0R, 2R/1R, 2R/2R, 3R/0R, 3R/1R, and 3R/2R (22, 23). Experimental data indicate that the number of VNTR repeats is inversely related to *XRCC5* expression, with an increase in the number of VNTR repeats linked to decreased *XRCC5* expression (27–29) (Figure 1A). VNTR polymorphisms in the *XRCC5* promoter are associated with sporadic bladder, gastric, and breast cancer (30–32).

Given the transmission pattern of VNTR, the uncertainty regarding the role of NHEJ in familial breast cancer, the presence of VNTR polymorphisms in the *XRCC5* promoter, and the association of VNTR polymorphisms with sporadic cancer, we hypothesized that VNTR in the *XRCC5* promoter could be involved in familial breast cancer. Therefore, we screened germline VNTR polymorphisms in the *XRCC5* promoter in three types of familial breast cancer (*BRCA1*^+^, *BRCA2*^+^, and *BRCAx*). The results showed that certain genotypes of VNTR polymorphisms are associated with the risk of familial breast cancer in *BRCA1*^+^ and *BRCA2*^+^ carriers.

## Materials and Methods

### Study Population

The familial breast cancer cases used in this study included three subtypes: familial breast cancer with *BRCA1* mutation (*BRCA1*^+^), familial breast cancer with *BRCA2* mutation (*BRCA2*^+^), and familial breast cancer without *BRCA1* or *BRCA2* mutations (*BRCAx*). Samples were obtained from the Hereditary Cancer Center at Creighton University (Tables S1–S3 in Supplementary Material). Healthy control samples of age- and gender-matched, de-identified Caucasian individuals were obtained from the Nebraska Biobank of the University of Nebraska Medical Center and The Nebraska Medical Center. The use of patient samples for this study was approved by the Institutional Review Board of Creighton University School of Medicine (00-12265) and of the University of Nebraska Medical Center (718-11-EP). Written and informed consent to participate in the study and to publicate the results was obtained from all subjects.

### Genotyping VNTR Polymorphisms in the *XRCC5* Promoter

PCR amplification, PAGE gel separation, and Sanger sequencing were used to determine VNTR genotype in the *XRCC5* promoter of each patient. PCR primer sequences were based on a previously published study (22) with sense primer 5′AGGCGGCTCAAACACCACAC3′ and antisense primer 5′CAAGCGGCAGATAGCGGAAAG3′. The PCR mixture consisted of DNA (20 ng), sense and antisense primers (10 pmol), and GoTaqH DNA polymerase (2 U, Promega). The PCR cycling conditions were 7 min at 95°C; 35 cycles of 30 s at 95°C, 30 s at 62°C, and 45 s at 72°C; and a final extension of 7 min at 72°C. An 8% PAGE gel was used to separate PCR products to determine allele type and genotype in each case (3R allele = 287 bp; 2R allele = 266 bp; 1R allele = 245 bp; and 0R allele = 224 bp). Representative products were isolated from PAGE gels and validated by Sanger sequencing.

### VNTR Genotypes in the *XRCC5* Promoter of Caucasians

Data from Iranian and Chinese healthy populations showed that VNTR genotypes in the *XRCC5* promoter can vary between ethnic groups (27, 28). To determine whether the data from these healthy populations can be used as suitable healthy controls for our study in breast cancer of Caucasian cases, we tested the genotypes of 100 healthy local Caucasian individuals and compared these with the genotypes from 535 Caucasian Iranian and 235 Chinese populations (27). The results showed no significant difference in genotypes between the local and Iranian Caucasian populations (*P* = 0.3774) with 2R/2R, 2R/1R, and 1R/1R as the major genotypes, but a significant difference was seen in the genotypes between local Caucasian and Chinese populations (*P* < 0.0001), and Iranian Caucasian and Chinese (*P* < 0.0001), whose genotypes included 2R/2R, 2R/1R, 2R/0R, 1R/1R, 1R/0R, and 0R/0R (Table 1). The 535 Iranian cases were from a Caucasian population living in the Fars province of Iran (27). Because these Iranian cases and our local cases were of the same ethnicity and there were no significant differences in genotypes between the two groups, the genotypes of the 100 local cases and the 535 Iranian cases were combined to make up the control population for downstream analyses. The combined control group is at Hardy-Weinberg equilibrium (*X*^2^ = 4.3485, df = 6, *P* = 0.6296).

**Table 1:** **Genotype distribution in three ethnical populations**.

Genotype	Local	Iranian	Chinese
3R/2R	0 (0)	4 (1)	0 (0)
3R/1R	1 (1)	8 (1)	0 (0)
3R/0R	1 (1)	1 (0)	0 (0)
2R/2R	16 (16)	84 (16)	28 (12)
2R/1R	50 (50)	205 (38)	57 (24)
2R/0R	5 (5)	29 (5)	71 (30)
1R/1R	22 (22)	168 (31)	12 (5)
1R/0R	5 (5)	33 (6)	37 (16)
0R/0R	0 (0)	3 (1)	30 (13)
Total	100 (100)	535 (100)	235 (100)
*P* value	Local to Iranian: 0.3774		
	Local to Chinese: <0.0001		
	Iranian to Chinese: <0.0001		

### Statistical Analyses

Fisher’s exact test was applied to determine the differences of VNTR polymorphism between the groups of familial breast cancer populations and control population, each type of breast cancer and cancer-affected and -unaffected subgroups within each type of cancer. Both odds ratios and their 95% confidence intervals and *P*-values were computed by using exact methods to keep consistency (33). Benjamini and Hochberg method was used to control the false positive rate at 0.05 (34). Analyses were performed using SAS^®^ software version 9.4 (SAS Institute Inc., Cary, NC, USA).

## Results

### Samples Used in the Study

*BRCA1*^+^ carrier refers to the women who tested positive for a pathogenic *BRCA1* mutation; *BRCA2*^+^ refers to the women who tested positive for a pathogenic *BRCA2* mutation; and *BRCAx* refers to the women who tested negative for the mutations in *BRCA1*, *BRCA2*, and *p53*, with two or more first or second degree relatives affected with primary *in situ* or invasive breast, ovarian, fallopian tube, or peritoneal cancer, and at least one person must have negative test result. Under each group, the cases were further divided into breast cancer (ovarian cancer)-affected and -unaffected carriers. The average ages at breast cancer diagnosis among the groups were 41.4 (*BRCA1*^+^), 43.6 (*BRCA2*^+^), and 47.7 (*BRCAx*). The age distributions are consistent with existing data that *BRCA1* and *BRCA2* mutation carriers tend to suffer cancer at earlier age. Most of the breast cancers were ductal type and ER-positive; all, but one, of the cases of ovarian cancer were invasive at diagnosis (Table 2).

**Table 2:** Summary of the *BRCA1*^+^, *BRCA2*^+^, and *BRCAx* carriers used in the study*.

Items	*BRCA1*^+^	*BRCA2*^+^	*BRCAx*
Unaffected cases	60	29	11
Average current age	56.9 ± 14.4	49.1 ± 14.4	66.4 ± 15.8
Affected cases	166	69	89
Average age at diagnosis	41.4 ± 10.8	43.6 ± 10.3	47.7 ± 12.0
Proband	38	15	62
Non-proband	128	54	27
Breast cancer	166	69	89
ER	22(+)43(−)	17(+)8(−)	27(+)6(−)
Unknown	101	44	56
PR	17(+)45(−)	15(+)9(−)	21(+)9(−)
Unknown	104	45	59
HER2/neu	4(+)10(−)	3(+)4(−)	6(+)17(−)
Unknown	152	62	66
Lymph nodes	38(+)54(−)	16(+)23(−)	16(+)16(−)
Unknown	74	30	57
Left	56	17	24
Right	55	25	27
Bilateral	40	19	8
Unknown	15	8	30
Adenocarcinoma not specified	28	7	17
Ductual carcinoma	89	43	59
Lobular carcinoma	4	4	4
Medullary carcinoma	24	3	
Mucoid or colloid carcinoma			3
Unknown	21	12	6
Invasive	148	63	79
*In situ*	5	2	3
Both invasive and *in situ*	6	3	3
Unknown	7	1	4
Ovarian cancer	21	5	15
Left			1
Right	3		
Bilateral	4	1	2
Unknown	14	4	12
Fallopian tube	1		1
Lymph nodes	7(+)10(−)	1(+)	2(+)3(−)
Unknown	4	4	10
Carcinoma, not specified	3		4
Clear cell adenocarcinoma	1	1	
Papillary adenocarcinoma	2	2	1
Adenocarcinoma (cystadenocarcinoma)	9	1	1
Endometrioid adenocarcinoma	2		
Serous (cyst)adenocarcinoma	5	1	3
Dysgerminoma			1
Unknown	1		5
Invasive	20	5	9
*In situ*	1		1
Unknown			5

*Some number in categories may not add up to the total due to incompleteness of tested cases.

### VNTR Genotypes in the *XRCC5* Promoter

The four VNTR alleles in the *XRCC5* promoter consist of three 21-bp (TGCGCATGCTCGGCGGGAATC) tandem repeats (3R), two 21-bp repeats (2R), one 21-bp repeat (1R), or without repeat (0R). The combination of PCR, PAGE, and Sanger sequencing methods provided an effective means to determine VNTR genotypes formed by the four alleles. Figure 1B shows the genotypes of homozygotes (1R/1R and 2R/2R) and heterozygotes (3R/2R, 3R/1R, 2R/1R, 2R/0R, and 1R/0R), and Figure 1C shows the sequences of the 21-bp repeats from the homozygotes (1R/1R and 2R/2R).

### VNTR Genotype Distribution, *BRCA* Predisposition, and Cancer Status

We compared the VNTR genotype distributions in the *XRCC5* promoter between three types of familial breast cancer: *BRCA1*^+^, *BRCA2*^+^, and *BRCAx* (Tables S1–S3 in Supplementary Material). The results show that the *BRCA1*^+^ and *BRCA2*^+^ groups differed significantly from the control group (*BRCA1*^+^ group: *P* < 0.0001; *BRCA2*^+^ group: *P* = 0.0042), but no difference was observed between the *BRCAx* groups and control group (*P* = 0.1308) (Table 3). To test whether different VNTR genotype distribution exists relating to disease status, the three types of familial breast cancer were divided into breast cancer-affected and breast cancer-unaffected subgroups and further compared each subgroup with the control group. The results show that the differences were only present between the cancer-affected subgroups in both groups of *BRCA1*^+^ (cancer-affected: *P* < 0.0001, cancer-unaffected: *P* = 0.2216) and *BRCA2*^+^ (cancer-affected: *P* = 0.0092, cancer-unaffected: *P* = 0.2748), but not in *BRCAx* (cancer-affected: *P* = 0.4251, cancer-unaffected: *P* = 0.5664) (Table 4). These results suggest the presence of association between VNTR genotypes and *BRCA1* and *BRCA2* mutation carriers affected with breast cancer.

**Table 3:** **Genotype distribution in three types of familial breast cancer**.

Genotype	Control	Familial breast cancer		
		*BRCA1*^+^	*BRCA2*^+^	*BRCAx*
Total	635 (100)	226 (100)	98 (100)	100 (100)
3R/2R	4 (1)	0 (0)	0 (0)	0 (0)
3R/1R	9 (1)	2 (1)	0 (0)	0 (0)
3R/0R	2 (0)	0 (0)	0 (0)	0 (0)
2R/2R	100 (16)	61 (27)	27 (28)	23 (23)
2R/1R	255 (40)	113 (50)	51 (52)	48 (48)
2R/0R	34 (5)	4 (2)	1 (1)	3 (3)
1R/1R	190 (30)	45 (20)	17 (17)	19 (19)
1R/0R	38 (6)	1 (0)	2 (2)	7 (7)
0R/0R	3 (0)	0 (0)	0 (0)	0 (0)
*P* value		<0.0001	0.0042	0.2185

**Table 4:** **Genotypes between cancer-affected and unaffected familial breast cancer**.

Genotype	Control	*BRCA1*^+^		*BRCA2*^+^		*BRCAx*	
		Cancer	No cancer	Cancer	No cancer	Cancer	No cancer
Total	635 (100)	166 (100)	60 (100)	69 (100)	29 (100)	89 (100)	11 (100)
3R/2R	4 (1)	0 (0)	0 (0)	0 (0)	0 (0)	0 (0)	0 (0)
3R/1R	9 (1)	2 (1)	0 (0)	0 (0)	0 (0)	0 (0)	0 (0)
3R/0R	2 (0)	0 (0)	0 (0)	0 (0)	0 (0)	0 (0)	0 (0)
2R/2R	100 (16)	44 (27)	17 (28)	17 (25)	10 (34)	20 (22)	3 (27)
2R/1R	255 (40)	85 (51)	28 (47)	39 (57)	12 (41)	41 (46)	6 (64)
2R/0R	34 (5)	3 (2)	1 (2)	0 (0)	1 (3)	3 (3)	0 (0)
1R/1R	190 (30)	32 (19)	13 (22)	13 (19)	4 (14)	18 (20)	1 (9)
1R/0R	38 (6)	0 (0)	1 (2)	0 (0)	2 (7)	7 (8)	0 (0)
0R/0R	3 (0)	0 (0)	0 (0)	0 (0)	0 (0)	0 (0)	0 (0)
*P* value		<0.0001	0.2216	0.0092	0.2748	0.4251	0.5664

We also compared the genotypes between the affected and unaffected subgroups in each group, and observed no difference in between (Table 5). We also evaluated the relationship between ages at diagnosis and VNTR polymorphism and observed no significant relationship in all three groups (data not shown). Therefore, there is no relationship between age of disease, cancer status, and VNTR polymorphism.

**Table 5:** **Comparison between affected and unaffected group**.

Genotype	Affected case	Unaffected case	Odds ratio	95% CI	*P*-value	Adjusted
***BRCA1***^+^						
2R/2R	45	16	0.9299	0.46–1.96	0.8637	0.9422
2R/1R	87	26	1.265	0.66–2.42	0.5403	1
1R/1R	32	13	0.7906	0.37–1.79	0.5664	1
1R/0R	0	1	0	0–64.08	0.2522	1
***BRCA2***^+^						
2R/2R	17	10	0.769	0.27–2.26	0.6281	0.9422
2R/1R	39	12	1.8417	0.70–4.91	0.1904	1
1R/1R	13	4	1.3929	0.38–6.45	0.7707	1
1R/0R	0	2	0	0–1.44	0.0854	1
***BRCAx***						
2R/2R	20	3	0.77	0.19–3.19	0.7118	0.9350
2R/1R	41	6	0.71	0.20–2.50	0.5951	1
1R/1R	18	1	2.54	0.30–21.12	0.6850	1
1R/0R	7	0	Infinity	0.23–infinity	1	1

### Specific Genotypes Associated with Risk of Familial Breast Cancer

Through comparing between control, breast cancer-affected and breast cancer-unaffected groups, we tested odds ratio to identify specific genotypes associated with risk of breast cancer (Table 6). Considering that the 3R and 0R groups contain only few cases in both control and carrier population, we removed 3R/2R, 3R/1R, 3R/0R, 2R/0R, and 0R/0R but focused on the 2R/2R, 2R/1R, 1R/1R, and 1R/0R as they contributed most of the cases. The results showed that

1. *BRCA1*^+^ group. 2R/2R (OR = 1.94, 95%CI = 1.26–2.95, *P* = 0.0096) and 2R/1R (OR = 1.58, 95%CI = 1.11–2.26, *P* = 0.0388) were associated with increased risk of breast cancer in cancer-affected *BRCA1*^+^ group, and 1R/1R (OR = 0.55, 95%CI = 0.35–0.84, *P* = 0.0196) and 1R/0R (OR = 0, 95%CI = 0–0.29, *P* = 0.0012) were associated with the decreased risk in cancer-affected *BRCA1*^+^ group;
2. *BRCA2*^+^ group. 2R/1R (OR = 1.94, 95%CI = 1.14–3.32, *P* = 0.0242) was associated with increased risk in cancer-affected *BRCA2*^+^ group. 2R/2R, 1R/1R, and 1R/0R had no association with the risk in cancer-affected *BRCA2*^+^ group;
3. *BRCAx* group. 2R/2R, 2R/1R, 1R/1R, and 1R/0R had no association with the risk of breast cancer in breast cancer-affected *BRCAx* group.

**Table 6:** **Association of VNTR genotypes in *XRCC5* promoter with familial breast cancer-affected and -unaffected groups**.

Genotype	Control	Affected case	Odds ratio	95% CI	*P*-value	Adjusted	Unaffected case	Odds ratio	95% CI	*P*-value	Adjusted
**BRCA1**^+^											
2R/2R	100	45	1.94	1.26–2.95	0.0016	0.0096	16	2.09	1.05–3.97	0.0221	0.1326
2R/1R	255	87	1.58	1.11–2.26	0.0087	0.0388	26	1.25	0.69–2.23	0.3190	0.4785
1R/1R	190	32	0.55	0.35–0.84	0.0049	0.0196	13	0.69	0.33–1.35	0.2906	0.4978
1R/0R	38	0	0	0–0.29	0.0001	0.0012	1	0.28	0.01–1.73	0.2414	0.5794
**BRCA2**^+^											
2R/2R	100	17	1.75	0.97–3.15	0.0865	0.1038	10	2.82	1.13–6.58	0.0174	0.2088
2R/1R	255	39	1.94	1.14–3.32	0.0101	0.0242	12	1.05	0.45–2.38	1	1
1R/1R	190	13	0.54	0.27–1.04	0.0680	0.0907	4	0.37	0.09–1.11	0.0632	0.1896
1R/0R	38	0	0	0–0.73	0.0427	0.0641	2	1.16	0.13–4.93	0.6916	0.7545
**BRCAx**											
2R/2R	100	20	1.55	0.90–2.67	0.1101	1	3	2.01	0.52–7.69	0.3945	0.5257
2R/1R	255	41	1.27	0.84–1.82	0.2882	0.3096	6	1.79	0.54–5.92	0.3654	0.5481
1R/1R	190	18	0.59	0.35–1.02	0.0583	0.3168	1	0.23	0.03–1.84	0.1885	0.5655
1R/0R	38	7	1.34	0.58–3.10	0.4913	1	0	0	0–4.75	1	1

## Discussion

Gene regulatory regions have long been considered a potential source of “missing heritability” in cancer (35, 36). Our study provides evidence showing that VNTR polymorphisms in the *XRCC5* promoter is associated with risk of familial breast cancer with *BRCA1*^+^ and *BRCA2*^+^ predisposition. Data from sporadic breast cancer showed that 2R/1R was not associated (OR = 1.09, 95%CI = 0.78–1.53, *P* = 0.595), but 0R/0R was associated with the disease (OR = 9.55, 95%CI = 1.19–76.6, *P* = 0.034) (31). The different results suggest that the association of VNTR polymorphisms in the *XRCC5* promoter differs between familial breast cancer and sporadic breast cancer.

For the *BRCA1*^+^ and *BRCA2*^+^ groups, the results can be explained by synergistic roles between Ku80 and *BRCA1/BRCA2* in maintaining genome stability through the NHRJ and HR pathways (37–39). Altered expression of Ku80 can disturb the synergy resulting in increased breast cancer risk in *BRCA* mutation carriers. The results also suggest that genotype 1R/1R and 1R/0R can reduce the risk of breast cancer in *BRCA1*^+^ carriers. Based on current knowledge, it is difficult to relate VNTR polymorphisms to *BRCAx* familial breast cancer, as genetic predisposition in this heterogeneous group of familial breast cancer remains to be determined. We did not observe a relationship between age at onset of disease, cancer status, and VNTR polymorphism. This could be due to the weaker influence by the VNTR polymorphism compared with that of the *BRCA* mutation predisposition. Alternatively, it could be due to the limited sample size used in the study, which restricts the statistical power to detect the potential significance. Further studies with larger sample size will help to address the issue.

In summary, our study indicates that 2R/2R and 2R/1R were significantly associated with increased risk, and 1R/1R and 1R/0R were significantly associated with the decreased risk of *BRCA1*^+^ breast cancer, whereas 2R/1R was significantly associated with the increased risk of *BRCA2*^+^ breast cancer.

## Author Contributions

JC and BD performed the genotyping and Sanger sequencing experiments; YK analyzed the genotyping data; JC and JL performed statistical analyses; CS, DB, and HL recruited breast cancer cases and extracted genomic DNA; and SW designed the study, interpreted the data, and drafted the manuscript. All authors read and approved the final manuscript.

## Conflict of Interest Statement

The authors declared that the research was conducted in the absence of any commercial and financial relationships that could be constructed as a potential conflict of interest.

## Abbreviations

*BRCA1*, breast cancer 1, early onset; *BRCA2*, breast cancer 2, early onset; *BRCAx*, familial breast cancer with wild type *BRCA1* and *BRCA2*; HR, homologous recombination; NHEJ, non-homologous end joining; PAGE, polyacrylamide gel electrophoresis; VNTR, variable number tandem repeat.
